# 

**Published:** 1804-06-01

**Authors:** 


### INDEX.

ABERDOUR, Dr. on vaccination 131
Ablution, on cold in ardent fever 21
———— and tepid, in fcarlatina 27
Abfcefs, on the method of opening an 327
Academicus, on a fatal diarrhœa 461
————, Mr. Cuming in anfwer to 518
Acid, on the poifonous qualities of pruffic 93
——, method of preparing the pure gallic 275
——, utility of muriatic 289
——, method of preparing arfenic 382
——, on treating paper with nitric 288
——, on combining antimony and muriatic 497
Acids, habitude of arfeniated hydrogen to 428
Aconite, its fuccefs in gout 225
Adye's, Mr. cafe of fracture 36
Affufion, on cold, in gout 521
Ague, on the benefits of white vitriol in 129, 246
——, cuprum ammoniacum recommended in 129
Aguft earth, account of the 285
Air, on contagious matter in the 82
—, on the means of purifying the 103
—, on preferving the falubrity of the 97
—, on the chemical changes produced by refpiration on 85
Albucafis on lithotomy *(note)* 4
Alkali, its effect in the bite of ferpents 332
————, volatile, its efficacy in poifon of ferpents 483
Alkaline falts, on fixed vegetable 464
Allen's, Mr. lectures annonnced 95
Almonds, cafes of children poifoned by bitter 92
Alpinus on extracting the ftone *(note)* 18
Amber, a large fpecimen of 287
Amaurofis fuccefsfully treated by electricity 135
Ambergris, analyfis of 167
America, on the population of 363
American epidemics, on 309
Amputation, reafons for deferring 68
————, advice on, in cafes of gun-fhot wounds 393
Anatomift's Vade-Mecum, review of the 189
Aneurifm of the aorta, cafe of 305
Aneurifm, cafe of 396
Angina maligna, utility of muriatic acid in 292
Anguftura, description and virtues of 566
Animal fluids, experiments on 287
Animals, on the puftulous eruptions of 431
————, on the action of opium on 145
————, on preferving the dead bodies of 358
————, effects of metals on the bodies of 287
Anftey, Mr. the author of an ode to Dr. Jenner 40
Antimony, on preparing tartrit of 497
Aorta, cafe of aneurifm of the 305
Apparatus altus, on the 5
Apples, on the fermentation of the juice of 415
Apoplexy, on emetics in 79
Arabic MS. on the fmall-pox, account of a 326
Arfeniated oxygen gas, on 402
Arfenic, its effects on withftanding putrefaction 192
————, its effect on animal bodies 287
————, internal ufe of in rheumatifm 492
———— acid, method of preparing the 382
Afh, virtues of the bark and leaves of the 435
Afpirators, defcription of the 101
Atkinfon's, Mr. obfervations on fevere dyfentery 508
Atmofphere, variety in the temperature of the 478
Atrophia ablactatorum, defcription of 185
Atrophy fuccefsfully treated by vaccination 252

Babington's, Dr. lectures announced 95
Baillie's, Dr. letter on ecchymofis of the labia 42
Bandage, account of an improved 304
Barlow, Mr. on the operation for the ftone 1
Bartlett's, Mr. cafe of hydrocephalus internus 301
Bartley, Mr. on delivering the placenta 456
Barton, Dr. on the bite of ferpents 334
Barytes, method of preparing muriated 382
Batavia, on the endemic of 201
Batty's, Dr. lectures announced 96
Beryll, new earth in the Saxon 285

Bilious complaints in warm climates, mode of treating 204

Bistoire cache, on the 14

Bladder, on wounds in the, *note*, 3

———, number of stones found in the 15

———, case of diseased 61

———, on the efficacy of hemlock in a case of diseased 342

Blagden, Mr. his case of ecchymosis of the labia 42

Blood, observations on carbon in the 47

——, on the changes produced by respiration on the 85

——, experiments on ox's 287

Bodies, animal, on preserving them from putrefaction 192, 358

Boger's lectures on the bones announced 192

Booker, Dr. on the cow pock 433

Books, critical analysis of medical—Richerand's Elements of Physiology, 84. Observations on the Antiphthisical Properties of the Lichen Islandicus, by R. Reece, 90. The London Practice of Midwifery, 91. Medical Ethics, or a Code of Institutes and Precepts, adapted to the professional Conduct of Physicians and Surgeons, by Thomas Percival, M.D. 181. Essays on the Diseases of Children, by John Cheyne, M.D. 184. The Anatomist's Vade Mecum, by Robert Hooper, M.D. 189. The Anatomy and Surgical Treatment of Inguinal and Congenital Hernia, by Astley Cooper, 279, 372. Observations on Pulmonary Consumption, or an Essay on the Lichen Islandicus, by J. B. Regnault, M.D. 283. A Synoptical Table of Diseases, by A. Crichton, M.D. 379. Researches into the Properties of Spring Water, by William Lambe, M.D. 462. Discourses on the Management of Infants and the Treatment of their Diseases, by John Herdman, M.D. 466. History of the Proceedings of the Committee appointed by the General Meeting of Apothecaries, Chemists, and Druggists in London, by William Chamberlaine, 474. An improved Method of treating Strictures in the Urethra, by Thomas Whately, 475, 558. Three Letters on Medical Subjects, by John Ford, M.D. 561. Elements of Materia Medica and Pharmacy, by J. Murray, 562. An Essay Medical, Philosophical, and Chemical, on Drunkenness, by T. Trotter, M.D. 568.

Books, new medical announced.—Boger's Lectures on the Diseases of the Bones, 192. Dr. Kinglake's Dissertation on Arthritis or Gout, *ib.* Dr. Trotter's Essay on Drunkenness, *ib.* Dr. Fothergill's Treatise on a singular Affection of the Nerves of the Face, *ib.* Mr. M'Gregor's Medical Sketches of the Expedition to Egypt, *ib.* Mr. Whately's improved Method of treating Strictures in the Urethra, 288. Dr. Routier's Considerations on the Puerperal Fever, 383. Mr. Parkinson's Examination of the mineralized remains of Vegetables and Animals of the Antediluvian World, 384. Dr. Kirby's Tables of the Materia Medica, 480. Dr. Harrington on Chemistry, 576.

Borichius, Prof. account of his death, *note*, 17

Botany, on the utility of 368, 439, 526

Bougies, on arming caustic 477

Braithwaite, Mr. on the cure of scarlet fever 289

Brooklime, on the virtues of 371

Brookes's, Mr. lectures announced 96

Brugnatelli, his Galvanic experiments 287

——— on nitric acid 288

Bryony, on the virtues of 447

Bubonocele, case of incarcerated 263

Buckholz, Mr. his method of preparing arsenic acid 382

Butterwort, on the virtues of 439

Calculous concretions, on, *note*, 2

Calomel its use in the croup 227

Camel pox, on the 326

Cancer uteri, on the method of operating for the 34

Cantharidum tinct. use of in hooping cough 191

Caoutchouc, description of the 398

Carbon in the blood, observations on 47

Carpue's, Mr. lectures announced 480

Cataract, on the membranous 70

Catarrh, on the Epidemic 488

Caustic, on, in strictures of the urethra 476

Celandine, its virtues in the venereal disease 139

Celsus on the operation for the stone 3

—— on cold water 151

Chamberlaine's History of the Proceedings of the Apothecaries, &c. reviewed 474

Chaussier on the blood 47

——— on preserving bodies from putrefaction 358

Chenevix, Mr. on the humours of the eye 163

——— on platina 288

Cherries, on the fermentation of the juice of 415

Cheyne, Dr. on the diseases of children reviewed 184

Chicken pox, observations on the 432

Children, on the diseases of 184

———, on the clothing of 470

———, two poisoned by bitter almonds 92

Cinchona, on the febrifuge principle of 215
———, experiments and obfervations on 266
Clarke's, Dr. lectures announced 96
Cleanlinefs, means of 97
———, obfervations on 469
Climates, on the difeafes of warm, 158, 200
Cline's, Mr. lectures announced 95
Cold affufion in gout, on 521
Coleman's, Mr. lectures announced 95
Coley's, Mr. cafe of anomalous vaccination 385
Colic, on the faturnine 463
Collot, the lithotomift, account of 9
Colon in the rectum, cafe of the invagination of the 115
Commiphora Madagafcarenfis, defcription of the *(with a plate)* 399
Confumption, digitalis fuccefsfully ufed in 286
Confumptive patients, directions to 288
Conftant reader on cold water in gout 150
———, Dr. Kinglake's reply to 241
Contagion of the influenza, remarks on 81
——— on variolous 83
———, on the means of preventing 97
———, benefits of nitrous vapour in preventing 136
Cooper's, Mr. anatomy and treatment of congenital and inguinal hernia reviewed 279, 372
——— lectures announced 95
Cork, an immediate conftituent of vegetables 288
Correfpondents, to 96, 288, 384, 480
Corrofive fublimate on preparing 340
Cortex falicis latifoliæ, on the genuine 400
Cow-pock inoculation, Mr. Cuftance on the 251
——— fucceeded by an eruption on the labia pudendi 385
———, on anomalous cafes of 388
Cow-pox, Dr. Booker on 433
———, Dr. Marfhall on 39
———, Mr. Morgan on 41
———, Mr. Cuftance on 251
Credulous in reply to Scepticus 154
Crichton's, Dr. Synoptical Table of Difeafes reviewed 379
Crotchet, on the ufe of the 496
Croup, on the treatment of the 227
Crump's, Mr. cafe of fiftula lacrymalis 551
Crural hernia, on 48, 119, 253
Cuming, Mr on gun-fhot wounds 388
——— on fphacelus 296
——— on white vitriol in agues 248
——— in anfwer to Academicus 518
Cuprum ammoniacum recommended in agues 129, 248
Curry's, Dr lectures announced 95
Cuftance's, Mr. cafe of vertigo and epilepfy 450
——— on vaccination 251
Cutaneous ulcerations, ufe of flores zinci in 93
Darnel, defcription and properties of 529
Dead bodies, effects of arfenic on 192
Debeck, Dr. on expelling the tape-worm 434
Denman, Dr. on the delivery of the placenta 65
Devonfhire, excellence of its climate 284
Dewar, Mr. on cold ablution in ardent fever 21
Diabetes mellitus, chemical analyfis of the urine in a cafe of 174
Diabetic urine, examination of the urine difcharged 338
Diaphragm, its agency in dilating the thorax 84
Diarrhœa, cafe of fatal 461
——— obfervations on that cafe 518
Digeftion, obfervations on 44
Digitalis in confumptions, on the ufe of 59, 286
———, on a tincture of 93
———, on its effects in phthifis 148
———, its fuccefs in incarcerated hernia 191
Difeafes of the bladder, on 61
———, fynoptical table of 379
———, account of, in an eaftern diftrict of London, from Nov. 20 to Dec. 20, 1803 83
———, from Dec. 20, 1803, to Jan. 20, 1804 180
———, from March 20 to April 20 478
——— from April 20 to May 20 557
Dog's grafs, defcription and properties of 530
Dropfy of the ovarium, on an encyfted 73
Drunkennefs, review of an Effay on 568
Duncan's, Dr. experiments on cinchona 266
——— on gum kino 278
Dyfentery, obfervations on fevere 508

Earth, account of a new 285
Ecchymofis of the labia, cafe of 42
Egineta on lithotomy 4
Egyptians, their method of extracting the ftone from the urethra *(note)* 18
Elaftic refin plants, defcription of 398
Electricity, its fuccefs in amaurofis 135
———, hiftory and theory of 523
Elements of Materia Medica and Pharmacy reviewed 562
Emetics recommended in yellow fever 337
——— tartar. injected into a vein 190
Emetics, on their ufe in apoplexy 79
England, on the climate of 284
———, on the population of 365
Epidemics, account of American 309
Epilepfy, cafe of 450
Epiftaxis, cafe of profufe 549

Eruptions, on herpetic (*with an engraving*) 230
———, on puftulous 431
Effays on the Difeafes of Children, reviewed 184
Excoriations, on the ufe of flores zinci in 93
Experiments, Galvanic, on the body of a criminal 94
——— on the humours of the eyes 163
——— on ambergris 169
——— on diabetic urine 174, 338
——— on cinchona 267
——— on gum kino 276
——— on the effects of arfenic on the human body 287
——— with Volta's pile on animal fluids 287
——— on meteorical fubftances 351
——— on muriated barytes 382
——— on fixed vegetable alkaline falts 408
——— on vinous fermentation 414
——— on arfeniated hydrogen gas 422
Extirpatio uteri, on the 34
Extra uterine pregnancy, cafe of 293
Eye, defcription of the 87
——, on the humours of the 163
——, cafe of a tumour of the (*with a plate*) 197

Fallopian tubes, on dropfy of the 73
Fauces, cafe of obftruction in the 190
Fees, on phyficians' 182
Fermentation, on vinous 411
———, on the theory of 421
Fever, on the remittent 201
———, on the cure of 289
———, on the virtues of cold ablution in 21
———, cafes of fcarlet 27
Fevers, on the effect of nitrous vapours in arrefting contagious 136
———, on the ufe of oil in 430
Fifher's, Mr. cafe of compound fracture 37
Fiftula lacrymalis, cafe of 551
Fluids, experiments on animal 287
Fogo, Mr. on the delivery of the placenta 63
——— on phthifis 147
Fontana, on the bite of ferpents 331
Ford's, Dr. three letters on medical fubjects, reviewed 561
Fothergill's, Dr. Treatife on a Diforder of the Face, announced 192
Fox glove, its fuccefs in hernia 191
Fracture, cafe of compound 37
——— of the fcull, cafe of 36
Franco on the operation for the ftone 5
Friction with oil, ufeful in fevers 480
Friefe, Dr. on digitalis in confumptions 286
Fungus hæmatodes, on the 210

Gallic acid, on preparing pure 275
Galvanic experiments 94
Galvanifm, its effects in hydrophobia 191
———, obfervations on 523
Gas, on arfeniated hydrogen 422
Gaftrotomy, on 118
Gelatine, not contained in cinchona 266
Gimbernat, M. on the crural hernia 124
——— on the prevention of contagion 136
Goldfon's pamphlet on vaccination, remarks on 505
Gonorrhœa in a child, cafe of 452
Goofeberries, on the fermentation of the juice of 413
Goofe grafs, defcription and properties of 533
Gooty, account of a difeafe called 431
Gorget, on the 13
Gout, fuccefsfully treated with hemlock 217
———, on cold water in 150
———, Mr. Taylor on 346
———, on regimen in 350
———, Dr. Kinglake on 241
———, on cold affufion in 521
Grape juice, on the fermentation of 415
Grafs root, defcription and qualities of 530
Gregory, Dr. on the death of 150
Gum kino, on the fubftance called 276
Gun-fhot wounds, on the treatment of 388

Hæmorrhagy, on the means of fuppreffing 68
Haighton's, Dr. lectures announced 96
Hall's Mr. cafe of gonorrhœa 452
Hardman, Mr. on opening an abfcefs 327
——— on the ufe of the crochet 496
Hare lip, new method of operating for the (*with a plate*) 193
Harrington's, Dr. Treatife on Chemiftry announced 576
Heart, on various morbid affections of the 305
Helm, Mr. on digeftion 44
Helmont, vindication of the character of Van 80
———, on the butter of antimony 497
Hemlock, efficacy of the extract of, in tetters 305
———, its benefits in gout 217
Hennen's, Mr. cafe of profufe epiftaxis 549
Hepatitis, common in warm climates 205
Herdman, Dr. on the Management of Infants, reviewed 466
Hernia, on crural 48, 119, 253
———, review of Mr. Cooper's Treatife on 279, 372
———, effects of digitalis in incarcerated 191
Herpetic eruptions, on 230

Hey, Mr. on the fungus hæmatodes 210
High operation for the ftone, on the 5
Hill, Mr. on opium 436
Hippocrates, the oath of 3
———— on cold water 151
Hoffman, on vegetable alkaline falts 404
Home, Mr. on the ftructure of the tongue 269
Hooper's, Dr. Anatomift's Vade-Mecum, reviewed 189
Hooping-cough, tinctura cantharidum recommended in 191
Hofpitals, on purifying the wards of 97
Hufeland, Prof. on the ufe of flores zinci in excoriations 93
———— on the hooping cough 191
———— on digitalis in hernia *ib.*
Hull, Dr. on crural hernia 48, 119, 253
Hume, Mr. on the pharmacopœia 298
———— reply to 534
Human ftomach, on the digeftive powers of the 44
Hydrocephalus internus, cafe of 401
Hydrogen gas, on arfeniated 422
Hydrophobia, effects of Galvanifm in 191
————, on fubmerfion in 459
————, on the application of opium in 539
Iceland mofs, on the properties of the 90, 283
Improvements in the medical art, on 360
Inebriety, on the effects of 160
Infants, on the management of 466
Influenza, on the contagion of the 81
———— on the 181, 488
Inoculation in meafles, on 459
Infect, defcription of a curious 451
Intermittents prevalent in China 20
———— on the cure of 246
Invagination, cafe of 115
Inverfio uteri, cafe of 207
Iron, on preventing the ruft of 286
Itch, ufe of the flowers of zinc in cafes of inveterate 93
Jaques, Frere, account of 9
Jaundice in infants, remarks on 184
Jenkinfon, Mr. on the cure of rheumatifm 492
Jenner, Dr. account of his picture 40
Jenner, Mr. on vaccination 552
Jennerian Society, account of the anniverfary of the Royal 572
Joints, on wounds of the 395

Kali purum cauftic, on its application in ftrictures 477

Kelch's, Mr. Galvanic experiments 94

Kelfon's, Mr. cafe of extra-uterine pregnancy 293

Keutfch, Dr. on the ufe of oil in fevers 480

Kine-pock, remarks on the 317

Kinglake, Dr. on digitalis 60

Kinglake, Dr. his work on the gout announced 192

———— the reply of Conftant Reader to 150

———— his anfwer 241

Kino, on the fubftance called 276

Kirby's, Dr. Tables of the Materia Medica, announced 480

———— lectures announced 96

Klaproth's, Mr. analyfis of diabetic urine 174

———— difcovery of a vegetable falt 288

———— on meteorical, ftony, and metallic fubftances 351

Labia, cafe of ecchymofis of the 42
Labia pudendi, cafe of vaccine vehicles on the 385
Lac fulphuris, remarks on the 437
Lambe, Dr. on the properties of fpring water 462
————'s, Dr. reply to the critique on his Refearches 570
Larynx, cafe of divided 61
Lateral operation, on the 9
Laurel water, pruffic acid in 93
Lead, its dangerous effects on water 462
————, on the diffolution of in water 570
Lectures announced. Meffrs. Cline and Cooper's on anatomy and furgery, 95. Drs. Babington and Curry on the practice of medicine, *ib.* Dr. Babington and Mr. Allen on chemiftry and experimental philofophy, *ib.* Dr. Curry on the theory of medicine and materia medica, *ib.* Dr. Haighton on midwifery and difeafes of women and children, *ib.* Ditto on phyfiology, *ib.* Mr. Cooper on furgery, *ib.* Mr. Coleman on the veterinary art, *ib.* Mr. Brookes on anatomy, phyfiology and furgery, 96. Dr. Clarke on midwifery, *ib.* Dr. Batty on midwifery and the difeafes of women and children, *ib.* Mr. Taunton on anatomy, phyfiology, and furgery, *ib.* Mr Syer on midwifery, *ib.* 288. Dr. Kirby on the principles and practice of medicine, 96. Mr. Carpue on anatomy and furgery, 480. Dr. Pearfon's on phyfic and chemiftry, 576. Mr. Saunders's on anatomy 576
Legrange's analyfis of ambergris 167
Lichen iflandicus, on the antiphthifical properties of 90
———— obfervations on 283
Lithotomy, hiftory and account of the operation of 3
Liver, on difeafes of the 205
Loffler's, Dr. cafe of inverfio uteri 207
London, account of difeafes in an eaftern diftrict of 83, 180, 478, 557
London Practice of Midwifery reviewed 91

Lungs, on the functions of the 46

Luxmoore's, Mr. improved bandage *(with plates)* 304

Maerker's, Dr. case of obstruction in the fauces 190

Mann, Abbé, his extraordinary case 217

Marianus, on the operation for the stone 5

Marshall, Dr. on vaccination 39

Marson, Mr. on the delivery of the placenta 328

Measles, on the 309

——— vulgar opinion on the origin of 432

——— on inoculation in the 459

Medical and Physical Intelligence 92, 190, 285, 382, 479, 568

——— act, on improvements in the 360

——— ethics, review of Dr. Percival's 181

——— lectures, see Lectures

——— speculations 458

Membranous cataract, on the 71

Metallic substance, account of a new 479

Metals, their action on the human body 287

Meteorical substances, analysis of 351

M' Gregor's, Mr. medical sketches announced 192

Midwifery, review of the London practice of 91

Miller, anecdote of a 570

Mitchill, Dr. on vegetable alkaline salts 404

Mongenot on vaccination 323

Monnot, Mr. on the membranous cataract 70

Moodie, Dr. on aneurism 305

———'s, Dr. case of a woman bitten by a viper 481

——— on the influenza 488

Morgan's, Mr. case of vaccination 41

Mortification, on preventing 296

Moffman, Dr. reply to 78

Mulberry, a saline mass exuded from the trunk of the white 288

Muriatic acid, its use in preventing contagion 105

——— its use in scarlet fever 289

Murray's Elements of Materia Medica and Pharmacy, reviewed 562

Naples, on the climate of 284

Naval surgeons, their hard case 395

Nice, city of, not favourable to consumptive people 284

Nicholas, M. his examination of diabetic urine 338

Nitre, its effects in preventing mortification 296

Nitric acid, its effects on paper 288

Nitrous vapours, their effects in preventing contagion 136

Obstruction in the fauces, case of 190

Oil, friction with, recommended in fevers 480

Opium, Mr. Ward on 111, 538

———, on the external application of, in hydrophobia 539

——— on the action of 145

——— Mr. Hill, on 436

——— on the exhibition of in wounds 361

Orchis, description and uses of 440

Ovarium, case of dropsy of the 73

Ox blood, experiment on 287

Oxen, account of the humours of the eyes of 166

Oxyd of antimony, on the preparation of 499

Oxydation of iron and steel, on preventing 286

Oxygen, on the quantity of heat combined with 46

Oxygenated muriatic acid, its use in scarlet fever 289

——— how to prepare 291

——— its effect in preventing contagion 292

Paper, on treating it with nitric acid 288

Paracelsus, on the alcahest of 497

Parkinson on the antideluvian world announced 384

Pears, on the fermentation of the juice of 415

Pearson's, Dr. lectures announced 576

Pearson, Mr. on the diseases of warm climates 200

Pelvis, on cases of distortion of the 496

Percival's, Dr. Medical Ethics, reviewed 181

——— on curing the orchis root 441

Pharmaceutical preparations, on 176

Pharmacopœia, errors in the 298

——— observations on the 534

Phifeldeck's, M. method of preparing corrosive sublimate 340

Phosphoric acid, crystallization of 177

Phosphorus, purity of 178

——— on white oxyd of 179

Phthisis, remarks on a case of 147

Physicians, on their professional assistance and fees 182

Physiology, elements of 84

Piepenbring, Dr. on the lac sulphuris 437

Placenta, on delivery of the 63, 328, 456

Plants, on the elastic resin 398

——— catalogue of British 368, 439, 526

Platina, experiments on 288, 479

Plica polonica, case of 316

Poison of serpents, on the 334, 482

——— of bitter almonds, case of 93

Population, on 156
——— of England, remarks on 365
Potter, Dr. on the epidemic difeafes of America 309
Poultry, difeafe peculiar to 431
Pregnancy, cafe of extra-uterine 293
Premature labour, cafe of 496
Privet, on the ufes of 370
Pruffic acid, its poifonous effects 93
Pudendi, cafe of vaccine veficles on the labia 385
Puerperal fever, a treatife on, announced 383
Pulmonary confumption, on digitalis in 60, 286
——— obfervations on 283
Purton, Mr. on the contagion of the influenza 81
Putrefaction, effects of arfenic in preventing 192
——— on preferving the bodies of animals from 358

Quack medicines to be difcouraged 183
Quacks and Empiricifm, account of 554
Quadrupeds liable to calculous concretions 2, *note*

Ramfay, Dr. on the bite of a fnake 382
Rattle-fnake, on the bite of the 385
Rectum, cafe of invagination of the colon in the 115
Reece's, Mr. obfervations on the lichen iflandicus, reviewed 90
Regnault's, Dr. obfervations on pulmonary confumption 283
Reid, Dr. on cold and tepid ablution in fcarlatina 27
Reig, Mr. on operating for the hare-lip (*with a plate*) 193
Renaud, Mr. on the effects of arfenic 287
Refearches into the properties of fpring water, reviewed 462
Refin tree, defcription of the 398
Refpiration, on the changes it produces on infpired air 85
Rheumatifm, on the cure of 492
Richerand's elements of phyfiology, reviewed 84
Ring, Mr. on vaccination 39
——— on the puftulous eruptions of animals 431, 553
Riverius, his character 80
Roche, Mr. on difeafed bladder 61
Rodman's, Mr. cafe of tumour of the eye (*with a plate*) 197
Rofes, cryftallization of the oil of 180
Routier, Dr. on puerperal fever 383
Roux's, Dr. cafe of invagination 115
Rufting of iron and fteel, to prevent 286

Saffron, defcription and virtues of 447
Salep, the excellent qualities of 441
Salmon's ftove ventilator defcribed 101
Salt, account of a new vegetable 288
Salts, on fixed vegetable alkaline 404
Saunder's, Mr. lectures announced 576
Saxon beryll, new earth difcovered in the 285
Scabious, defcription and qualities of 531
Scarlatina, fuccefsfully treated with cold and tepid ablution 27
Scarlet fever, on the ufe of muriatic acid in 289
Scepticus, reply to 154
Scheele's, Mr. method of preparing butter of antimony 497
Schnaubert's method of preparing pure gallic acid 275
Scott, Dr. on white vitriol in agues 129
——— anfwer to 248
Scull, cafe of fractured 36
Sea matweed, defcription and ufes of 526
Seamen, on their hardy conftitution 569
Seguin, on the febrifuge principle of cinchona 215
——— theory of fermentation 421, *note*
Seneca, or fnake-root, its beneficial qualities 335
Serpents, on the bite of 384
———, obfervations on the poifon of 482
Sheep's eyes, analyfis of 164
Ships, on ventilating 201
Small-pox, its prevalence in the metropolis 85
——— anfwer to Scepticus on the danger of exterminating the 154
——— on the extermination of the 362
——— mifcellaneous remarks on the 317
——— on the origin of the 326
——— on the extinction of the 460
Smith's, Mr. cafe of amaurofis fuccefsfully treated by electricity 135
Snakes, on volatile alkali as a cure for the bite of 382
Specifics, on 129
Speedwell, virtues of 371
Sphacelus, cafes of 296
Steel, how to prevent the rufting of 286
Steinacher, M. on pharmaceutical operations 176
Stomach, on the digeftive power of the human 44
Stone, practical obfervations on the operation for the 1
Stott, Mr. on preparing tartrit of antimony 497
Stove ventilator, defcription of the 101
Struve, Dr. on the cancer uteri 34
Sublimate, new method of preparing corrofive 349

Submerfion in hydrophobia, on 459
Sugar, action of ferment on 418
Sulphuris, remarks on the lac 437
Surgeons, advice to naval 395
——— hofpital, caution to 106
Suttons, their practice of inoculation 155
Swine, meafles vulgarly fuppofed to originate in 432
Syer's, Mr. lectures announced 96

Tape-worm, new method of expelling the 434
Tartrit of antimony, on the preparation of 497
Taunton's, Mr. lectures announced 96
Taylor, Mr. on cold water in gout 346
Temperament, remarks on 89
Tetanus, on opiate frictions in 539
———, on opium in 539
Tetters, on the extract of hemlock in the cure of 342
Thenard, Cit. on vinous fermentation 411
Thomas, Dr. on the functions of the lungs 46
Thorax, on the dilatation of the 84
Tice, Dr. on the cure of intermittents 246
Tilefius, Prof. on herpetic eruptions 230
Tongue, on the ftructure of the 269
Tromfdorff's method of preparing muriated barytes 382
——— on arfeniated hydrogen gas 422
Trotter's, Dr. Effay on Drunkennefs announced 192
——— Effay, review of 568
Tumour of the eye, cafe of 197
Tynicola in reply to Dr. Moffman 78

Vaccination, Dr. Marfhall on 39
——— Mr. Morgan's cafe of 41
——— Dr. Aberdour's cafes of 131
——— Mr. Jenner on 552
——— Walker's remarks on Goldfon's pamphlet on 505
Vaccine-pock inftitution, annual meeting of the 573
Vaccine veficles on the labia pudendi 385
Vagina, on difcharges from the 453
Valentine, Dr. on the efficacy of hemlock in tetters 342
Valerian, on the virtues of 445
Vander Heylen on cold water 151
Vauquelin, Cit. experiments on gum kino 276
——— on a new metallic fubftance 479
Vegetable alkaline falts, obfervations on 404
——— falt, difcovery of a new 288
Vegetables, cork one of the conftituents of 288
Venereal difeafe, efficacy of celandine in 139
Ventilator, defcription of a ftove *(with a plate)* 101
Ventriloquifm, remarks on 89
Vertebræ, on wounds of the 361
Vertigo, cafe of 450
Vinegar, on the ufe of, in preventing contagion 104
———, on the ufe of, on board fhips 510
Vinous fermentation, on 411
Viper, on the bite of the 337
——— cafe of a woman bitten by a 481
Vitriol, white, on its ufe in agues 129, 248
Voifier's, Mr. cafe of encyfted dropfy of the ovarium and Fallopian tubes 73
Volatile alkali, its fuccefs in the bite of fnakes 332
Volta's pile, experiments with, on animal fluids 287

Underwood, Dr. on difcharges from the vagina, with remarks 453
Unguentum nitratum, formula for 176
Urethra, quantity of ftones extracted from the 15, *note*
——— on treating ftrictures in the 475, 558
Urine, chemical analyfis of 174
——— experiments on diabetic 338
Uteri, cafe of inverted *(with an engraving)* 207
Uterus, cafe of difplacing the 73

Walker's, Dr. remarks on Goldfon's pamphlet on vaccination 505
Ward, Mr. on opium 111, 538
Wards in hofpitals, advice concerning 107
Water, on cold in the gout 150, 346
———, refearches into the properties of fpring 462, 570
Waterflag, on the virtues of 448
Wawn, Mr. on the treatment of the croup 227
Weaning brafh in children, remarks on 185
Welper, Dr. on the effects of arfenic 192
Wendt, Prof. on the ufe of celandine in the venereal difeafe 139
Weftcote, Mr. on fuppreffing hæmorrhagy 68
Whately, Mr. on treating Strictures in the Urethra, reviewed 475, 558
White's Mr. cafe of aneurifm 396
Wildenow, Prof. on the elaftic refin plants *(with a plate)* 398
Wilkinfon, Mr. on the cortex falicis latifoliæ 400
Wilfon, Dr. on the action of opium on living animals 145
Willow, defcription and ufe of the 443
Winterbottom, Dr. in reply to Dr. Moffman 78

Women, on extracting the ftone from 16
Woodruff, defcription and qualities of 531
Wounds in the bladder, on 3
———, on gun-fhot 388

Yellow fever, analogy between the poifon of ferpents and 337

Younglove, Dr. on the fmall-pox and king pock 317

Zinc, flowers of, ufeful in excoriations and cutaneous eruptions 93
———, on the preparation and application of 139

### REFERENCES TO THE PLATES.

Mr. Adye's Sketch of a remarkable thin Cranium — — 1
Dr. Struve's Instrument for operating for the Cancer — — *ib.*
Mr. Barlow's Instrument for the Stone — — — — *ib.*
The Stove Ventilator — — — — — — — — 97
Mr. Rieg on the Hare Lip — — — — — — — 193
Mr. Rodman's Case of Tumour of the Eye — — — 197
Dr. Loffler's Case of Inversio Uteri — — — — — *ib.*
Prof. Tilesius on Herpetic Eruptions — — — — 230
Mr. Luxmoore's Improved Bandage — — — — — 304
Commiphora Madagascarensis & Siphonia Cuchucu — 398

